# Prevalence and antibiotic susceptibility patterns of *Shigella* and *Salmonella* among children aged below five years with Diarrhoea attending Nigist Eleni Mohammed memorial hospital, South Ethiopia

**DOI:** 10.1186/s12887-018-1221-9

**Published:** 2018-07-25

**Authors:** Wondwossen Abebe, Alemu Earsido, Solomon Taye, Mesfin Assefa, Adane Eyasu, Girma Godebo

**Affiliations:** 1 Department of Medical Microbiology, School of Biomedical and Laboratory Sciences, College of Medicine and Health Sciences, University of Gondar, Gondar, Ethiopia; 2 Department of Public Health, College of Medicine and Health Sciences, Wachemo University, Hossana, Ethiopia; 3 Department of Medical Laboratory Science, College of Medicine and Health Sciences, Wachemo University, Hossana, Ethiopia

**Keywords:** *Shigella*, *Salmonella*, Diarrhoea, Children aged below 5 years, Antibiotic susceptibility, Hosanna, Ethiopia

## Abstract

**Background:**

Diarrhoeal disease is the second leading cause of death among children aged below 5 years. Even though, both preventable and treatable diseases, globally there are nearly 1.7 billion cases of childhood diarrhoeal disease and responsible for killing around 525,000 children every year. *Shigella* and *Salmonella* species were the leading cause of etiologic agents for diarrhoea associated deaths. The aim of this study was to determine the prevalence and antibiotic susceptibility patterns of *Shigella *and *Salmonella *isolated from children aged below 5 years with diarrhoea attending Nigist Eleni Mohammed Memorial Hospital, Hossana, South Ethiopia.

**Methods:**

A cross sectional study was conducted from June 02 to September 24, 2017. Two hundred four children aged below 5 years with diarrhoea were enrolled consecutively using convenience sampling technique. Stool specimens were processed in accordance with the standard bacteriological methods and antibiotic susceptibility pattern of the isolates was determined using disc diffusion method. Data were analyzed using SPSS version 20.

**Results:**

Out of the 204 children aged below 5 years with diarrhoeal disease 19/204 (9.3%, [95%CI, 5.7–13.7%]) of them were positive for bacterial growth, of which 17/204(8.3%) were *Shigella* species and 2/204(1%) were *Salmonella* species*.* Both *Shigella* and *Salmonella* isolates were 100% susceptible to norfloxacin, nalidixic acid and kanamycin. However, isolates of *Shigella* showed 100, 76.5 and 64.7% resistance to ampicillin, gentamicin and cotrimoxazole respectively while *Salmonella* species were highly resistant to ampicillin and gentamicin (100% each).

**Conclusions:**

*Salmonella* and *Shigella* species is prevalent in the current study area. Among the tested antibiotics, norfloxacin, nalidixic acid and kanamycin were found to be most effective for both isolates. Both species are developing resistance to the commonly prescribed antibiotic. Therefore, culture based bacterial species identification and antimicrobial susceptibility testing services are strongly recommended to avoid empirical treatment in the study area.

## Background

Diarrhoeal disease is the second leading cause of death among children aged below 5 years. Even though, both preventable and treatable diseases, globally there are nearly 1.7 billion cases of childhood diarrhoeal disease and responsible for killing around 525,000 children every year. The burden of diarrhoeal disease is highest in developing country where there is poor sanitation, inadequate hygiene, unsafe drinking water, as well as poorer overall health and nutritional status [1].

A wide variety of aetiological agents are responsible for causing diarrhea such as *Shigella spp* (Shigellosis)*, Vibro cholera* (Cholera), typical enteropathogenic *Escherichia coli* (tEPEC*),* entrotoxigonic *E. coli (*ETEC*),* non-typhoidal *Salmonella spp*, *Clostridium difficile*, *Aeromonas spp*, *Campylobacter spp,* (*Campylobacter* enteritis)*,* Rotavirus (Rotaviral enteritis), enteric Adenovirus (serotype 40 and 41)*,* Norovirus*, Entamoeba histolytica* (Amoebiasis), and C*ryptosporidium spp* (Cryptosporidiosis). Among these agents Rotavirus, *Shigella spp* and *Salmonella spp* are the leading cause of diarrhea deaths [2].

In recent years the emergence and global dissemination of *Salmonella* and *Shigella* species resistant to ampicilline, chloramphenicol, tetracycline and co-trimoxazole (trimethoprime-sulphamethoxazole) has been increasingly documented in developing countries [3]. Inappropriate antibiotic use and limited laboratory facilities to test antimicrobial susceptibility has led to an increased antimicrobial resistance and reduced therapeutic efficacy in the developing countries [4].

In Ethiopia, diarrhea is one of the major contributors to deaths for children aged below 5 years and contributes to more than one in every ten (13%) child deaths. The prevalence of diarrhoea is high (12%) in children aged below 5 years. However, it is relatively higher among children aged from 6 to 23 months [5]. Furthermore, isolates of *salmonella* and *shigella* showed different prevalence rates and high rate of drug resistance to the commonly used antibiotic agents in different regions [4, 6–9]. There is no published data from the study area (Hosanna town) on the prevalence and antimicrobial susceptibility patterns of *salmonella* and *Shigella* among diarrhoeic children aged below 5 years. Therefore, this study aimed to fill the existing information gap in the current study area.

## Methods

### Study design, area and population

A cross sectional study was carried out from June 2017 to September 2017 on children aged below 5 years with diarrhea attending Nigist Eleni Mohammed Memorial Hospital (NEMMH), Hosanna town, South Ethiopia. Hosanna town is located 232 km southwest of Addis Ababa, the capital city of Ethiopia. The hospital is located in Hosanna city and is the only hospital in the area. Currently, the hospital provides health services to more than 2.3 million people residing in Hadiya and Kembata Tembaro zones. It has 88 beds for inpatient services in four wards (medical, pediatric, surgical and gynecology/obstetrics). The hospital is also provides outpatient health services. The Hosanna branch of regional health laboratory is also found in the hospital compound providing its services.

### Sample size and sampling technique

The sample size was calculated based on the assumption of 5% expected margins of error and 95% confidence interval, taking the prevalence of 15% from the previous study which was conducted in Butajira town, Ethiopia on diarrheal patients [9] using a single population proportion formula (9) as follows.

$$ \mathrm{n}=\frac{{\left(\mathrm{Z}\upalpha {/}_2\right)}^2\mathrm{P}\left(1\hbox{-} \mathrm{P}\right)}{{\mathrm{d}}^2} $$

Where:

n = calculated sample size.

Z = standard normal deviate at 95%, Confidence Interval = 1.96.

P = prevalence from the previous study = 15%.

d = precision level = 0.05.

The calculated sample size was 196, adding 5% non-response rate, the total calculated sample size was 206 study participants. The study participants were enrolled consecutively using convenience sampling technique until a sample size of 206 study participants was achieved. All study participants were affected by diarrhea (diarrhea defined as the passage of three or more loose or liquid stools per day) [1]. The physicians collected socio-demographic information and other required information like antibiotic taken before data collection from their parents/guardians. However, children who had taken antibiotic within 7 days before data collection, those who were aged above 5 years and those children whose parents/guardians did not consent to participate in the study were excluded from the study. The aims of the study and benefits of participation were clearly explained for the participants prior to data collection. Participation was on voluntarily basis and they were told that it was within their right to withdraw from the study at any time in the course of the study.

### Specimen collection and cultural identification

Freshly passed stool was collected, placed immediately in Cary Blair transport medium (Oxoid Ltd., Basingstoke, UK) and then transported to the Hosanna branch of regional laboratory within 3 hours of collection for further processing. The specimens were placed in Selenite F enrichment broth (Oxoid, UK) and incubated at 37 °C for 24 h. And then sub-cultured onto deoxycholate agar (DCA) and xylose lysine deoxycholate agar (XLD,) (Oxoid UK) agar and incubated at 37 °C for 18–24 h. The growth of *Salmonella* and *Shigella* species was detected by its colony characteristic appearance on XLD agar (*Shigella*: red colonies, *Salmonella*: red with a black centre) and DCA (*Shigella*: pale colonies, *Salmonella*: black center pale colonies). The suspected colonies were further tested through a series of biochemical tests to identify *Shigella* and *Salmonella* species [10].

Disk diffusion technique was performed to assess the antibiotic resistance / susceptibility pattern of *Salmonella* and *Shigella* isolates. The antibiotic susceptibility testing of all strains were carried out on Muller-Hinton agar (Oxoid, UK) with antibiotic discs (Oxoid, UK) using the single disc diffusion technique against ampicillin (10 μg), co-trimoxazole, (trimethoprim/sulphamethoxazole) 1.25/23.75 μg), chloramphenicol (30 μg), ciprofloxacin (5 μg), ceftriaxone (30 μg), nalidixic acid (30 μg), gentamicin (10 μg), norfloxacin (10 μg) and kanamycin(30 μg) based on the Standard Operating Procedure (SOP) adapted from Clinical and Laboratory Standards Institute (CLSI 2017 edition) and results were reported as sensitive, intermediate and resistance. To standardize the inoculums density for a susceptibility test, a BaSO4 turbidity standard, equivalent to a 0.5 McFarland standard was used strictly following the SOP for the preparation and standardization [11]. Multiple drug resistance is defined as the resistance of an isolate to two and more drugs within one class of drug [9].

### Data analysis and interpretation

Data were entered and analyzed using SPSS version 20 software. Results were presented through graphs and tables. Statistical significance of association was measured by using Chi-square test. A *p*-value < 0.05 was considered as statistically significant.

## Results

### Socio-demographic characteristics

**Fig. 1:**
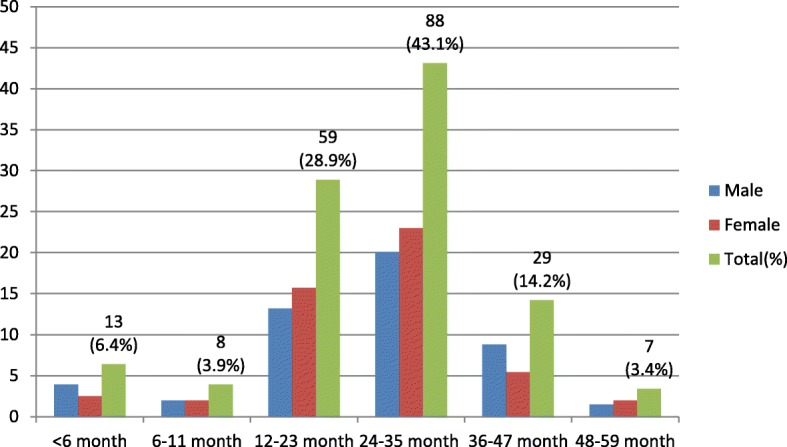
Distribution of participants by age and sex

A total of 204 children aged below 5 years with diarrhoea were included in the study. Out of the 204 study participants, 103/204 (50.5%) were females. The ages of the study participants ranged from 2 to 59 months with a mean of 26.2 months (SD ±11.457): 88/204 (43.1%) of them were between 24 and 35 months, 59/204(28.9%) were between 12 and 23 months, and 29/204(14.2%) were between 36 and 47 months old (Fig. 1). Two study participants were excluded due to insufficient sample provision for laboratory investigation.

### Prevalence of *Shigella* and *Salmonella*

Of the 204 children aged below 5 years with diarrhoeal disease 19/204 (9.3%, [95% CI, 5.7–13.7%]) revealed bacterial growth, of which 17/204(8.3%) were *Shigella* species and 2/204(1%) were *Salmonella* species*.* The frequency of isolation of *Shigella* species was highest among the age group of 24 and 35 months, furthermore, the only two *Salmonella* isolates were encountered among 12–23 month old, but has no statistical significance with the age interval. Among the 19 culture positive children, 11/19(57.9%) were females (Table 1).

**Table 1:** Distribution of *Shigella* and *Salmonella* by age and sex isolated from children aged below five years with diarrhea at NEMM Hospital, Hosanna town, South Ethiopia, 2017

Variable	*Salmonella* spp. (*n* = 2)		*Shigella* spp. (*n* = 17)		Total positive (*n* = 19) No (%)	*P*-value
	Positive No (%)	Negative No (%)	Positive No (%)	Negative No (%)		
Age(month)						
< 6	0 (0)	13 (100)	0 (0)	13 (100)	0 (0)	0.181
6–11	0 (0)	8 (100)	1 (12.5)	7 (87.5)	1 (5.3)	
12–23	2 (3.4)	57 (96.6)	3 (5.1)	56 (94.9)	5 (26.3)	
24–35	0 (0)	88 (100)	10 (11.4)	78 (88.6)	10 (52.6)	
36–47	0 (0)	29 (100)	2 (6.9)	27 (93.1)	2 (10.5)	
48–59	0 (0)	7 (100)	1 (14.3)	6 (85.7)	1 (5.3)	
Sex						
Male	1 (1)	100 (99)	7 (6.8)	94 (91.2)	8 (42.1)	0.811
Female	1 (1)	102 (99)	10 (9.7)	93 (90.3)	11 (57.9)	

### Antibiotic susceptibility test patterns

Among all antibiotic tested, all isolates of *Shigella* spp. were susceptible to norfloxacin 17/17(100%), nalidixic acid 17/17(100%), and kanamycin 17/17(100%) while, 14/17(82.4%) Strains were resistant to ampicilin, 13/17(76.5%) to gentamicin, and 11/17(64.7%) to co-trimoxazole. *Salmonella* isolates were also susceptible to norfloxacin, nalidixic acid, kanamycin, chloramphenicol, ciprofloxacin and ceftriaxone, but 100% resistant to gentamicin and ampicillin (Table 2).

**Table 2:** Antibiotic resistance patterns of *Shigella* and *Salmonella* isolate among children aged below 5 years with diarrhoea at NEMM Hospital, Hosanna town, South Ethiopia, 2017

Isolate	No (%) of isolates resistance									
	n	AMP	GEN	KAN	NAL	CRO	SXT	CAF	NOR	CIP
*Shigella*	17	14 (82.4)	13 (76.5)	0 (0)	0 (0)	3 (17.6)	11 (64.7)	8 (47.1)	0 (0)	3 (17.6)
*Salmonella*	2	2 (100)	2 (100)	0 (0)	0 (0)	0 (0)	1 (50)	0 (0)	0 (0)	0 (0)
Total	19	16 (84.2)	15 (78.9)	0 (0)	0 (0)	3 (15.8)	12(63.2)	8 (42.1)	0 (0)	3 (15.8)

Key: *AMP* Ampicillin, *TTC* Tetracycline, *GEN* Gentamicin, *KAN* kanamycin, *NAL* Nalidixic acid, *CRO* ceftiraxone, *SXT* Trimethoprime-Sulphamethoxazole(co-trimoxazole), *CAF* Chloramphenicol, *NOR* Norfoloxacin, *CIP* Ciprofloxacin

The overall multiple drug resistance patterns were 12/19(63.2%) while none of them was sensitive to all antimicrobial drugs tested. Of the seventeen *Shigella* isolates 11/17 (64.7%) were resistant to more than 3 antimicrobials, In addition among the two isolates of *Salmonella* spp. one was found to be resistant to more than 3 antibiotics (Table 3).

**Table 3:** Antibiogram of S*higella* and S*almonella* isolated among children aged below 5 years with diarrhoea in NEMMH, Hosanna town, South Ethiopia, 2017

Isolate	Antibiogram				
	No. of isolates	R_0_ No (%)	R_1_ No (%)	R_2_ No (%)	≥R_3_ No (%)
*Shigella*	17	1 (5.9)	1 (6.2)	4 (26.7)	11 (64.7)
*Salmonella*	2	0	0	1 (50)	1 (50)
Total	19	5.3	5.6	29.4	63.2

*R1* Resistance for one drug, *R2* Resistance for two drugs, *≥ R3* Resistance for three and above drugs

## Discussion

The isolation rate of *Shigella* (8.3%) in our study was comparable to previous studies in Ethiopia: Addis Ababa, 9.1% [12], Hawassa, 7% [13], Gondar, 7.5% [14], 8.7% [15], Bahir Dar, 9.5% [16] and Mekelle, 6.9% [17]. However, it is lower than other findings in Hawassa [5], Jimma [18], Harar [19], Gondar [4] and Bahir Dar [20] with the isolation rate 34.6, 20.1, 14.6, 16.9 and 14.9% respectively. Our finding is higher than studies conducted in other part of Ethiopia: Butajira, 4.5% [9], Jimma, 2.3% [21], 1.1% [22] and Gondar, 4.6% [23]. These variations may be due to differences in study participant, study period and increased awareness of the community about personal and environmental hygiene made by the health extension workers being implemented by the government and by health science students from Wachamo University during their field practice. Furthermore, there is no research conducted around Hosanna town on identification and characterization of *Shigella* isolates to see prevalence variation over time.

In the current study, the isolation rate of *Salmonella* (1%) was found to be lower than other studies done in Ethiopia at different areas: Addis Ababa, 3.95% [12], Butajira, 4.5% [9], Harar, 11.5% [8], Jimma, 15.4% [18] and Bahir Dar 7.8% [16]. However, it was consistent with the findings reported in Addis Ababa, 0% [24], Hawassa 0% [25], 2.5% [13], Gondar 1.6% [26], 1.1%(23). This could be due to difference in study participant, study period, geographical and seasonal variation, and increased awareness of the community about personal and environmental hygiene. Better awareness of the community especially mothers about their personal and environmental hygiene directly influence the prevalence of *Shigella* and *Salmonella* among their children.

Norfloxacin, nalidixic acid and kanamycin were 100% effective against *Shigella* while there was a resistance for ampicillin (82.4%), gentamicin (76.5%) and co-trimoxazole (64.7%) which is comparable with previous report from Gondar, where 100 and 93% of the isolates were sensitive to norfloxacin and kanamycin respectively [15]. In addition, in similar study conducted in Gondar, Ethiopia, *Shigella* isolates showed comparable pattern of high resistance against ampicillin (78.9%) and co-trimoxazole (84.6%) and lower resistance to gentamicin (12.2%), ciprofloxacin (2.2%) and norfloxacin (1.1%) [14].These findings indicate that treatment needs to be based on species identification and susceptibility testing rather than the currently practiced empirical treatment [8, 24].

The high level of antibiotic susceptibility of *Salmonella* to chloramphenicol, ciprofloxacin and norfloxacin is in agreement with earlier studies reported from Ethiopia [9, 21]. The resistance of *Salmonella* towards ampicillin (100%) was similar to studies from Jimma, 100% [22] and Harar, 100% [8]. High resistance was also observed to gentamaycin (100%) which agrees with reports Addis Ababa [4]; in contrast to low resistance report from Jimma 5.2% [22] and Harar 7.2% [8]. The rise in resistance might be due to selective pressure created by the use of antimicrobials in food processing animals and irrational use of antibiotics [27].

In present study, over 63% (12/19) of the isolates were resistance two or more antibiotics and none of the strains were sensitive to all antimicrobials tested. Of the seventeen *Shigella* isolates, 11/17 (64.7%) were resistant to more than 3 antimicrobials. This shows that antibiotics remain the most important therapy for successful bacterial infections; however these inexpensive and widely available antimicrobials can no longer be used empirically [3, 17].

## Conclusions

*Salmonella* and *Shigella* species is prevalent in the current study area. Among the tested antibiotics, norfloxacin, nalidixic acid and kanamycin were found to be most effective for both isolates. Both species are developing resistance to the commonly prescribed antibiotic. Therefore, culture based bacterial species identification and antimicrobial susceptibility testing services are strongly recommended to avoid empirical treatment in the study area.

## Abbreviations

DCA: Deoxycholate Agar
NEMMH: Nigist Eleni Mohammed Memorial Hospital
SOP: Standard Operating Procedure
XLD: Xylose lysine deoxycholate agar
